# Association between periodontal status and salivary interleukin-6 levels in normal-weight and obese individuals with and without prediabetes

**DOI:** 10.1038/s41598-026-49503-1

**Published:** 2026-04-25

**Authors:** Marwa Y. Shaheen, Amani M. Basudan, Rakan Saifuddin Shaheen, Fatemah M. AlAhmari, Nouf Alshibani, Lamees Alssum, Fatima Alzahraa Yassin Shaheen, Mohammed Rami Shaath, Reem Al-kattan

**Affiliations:** 1 Department of Periodontics and Community Dentistry, College of Dentistry, King Saud University, P.O. Box 2455, Riyadh, 11451 Saudi Arabia; 2 Periodontics Division, Department of Preventive Dentistry, College of Medicine and Dentistry, Riyadh Elm University, Riyadh, Saudi Arabia; 3 Consultant, Emergency department, SFHPR security force hospital program, Riyadh, Saudi Arabia; 4 Department of Medicine, College of Medicine, Alfaisal University, Riyadh, Saudi Arabia

**Keywords:** Alveolar bone loss, Clinical attachment loss, Interleukin-6, Obesity, Prediabetes, Saliva, Biomarkers, Diseases, Endocrinology, Health care, Medical research

## Abstract

The objective was to investigate the association between periodontal status and whole salivary interleukin-6 (IL-6) concentrations in normal-weight and obese individuals with and without prediabetes. Normoglycemic and prediabetic adults with obesity and systemically healthy individuals (controls) were encompassed. Participants were alienated into four-groups: Group-1: individuals with prediabetes alone, Group-2: individuals with obesity alone, Group-3: prediabetic individuals with obesity, and Group-4: systemically healthy individuals. Patient demographics were recorded. Clinical evaluations comprised measurements of probing depth (PD), clinical attachment loss (CAL), marginal bone loss (MBL), gingival index (GI), and plaque index (PI). Hemoglobin A1c was recorded and whole salivary IL-6 levels were measured. Associations between IL-6 levels, demographic variables, and periodontal parameters were examined using linear regression analysis. Intergroup differences were evaluated, and a significance threshold of *P* < 5% was applied. One-hundred-and-four individuals were assessed for eligibility. Fifteen individuals were excluded. In total, 89 individuals were included. The PI (*P* < 0.01),GI (*P* < 0.01),CAL (*P* < 0.01),PD (*P* < 0.01), MBL (*P* < 0.01),MT (*P* < 0.01) and IL-6 levels (*P* < 0.01) were significantly higher among individuals in Group 3 than groups 1, 2 and 4. There was a significant correlation between PD and IL-6 levels in group-3 (*P* < 0.001). Prediabetes in the presence of obesity is associated with an increased burden of periodontal inflammation; however, this relationship appears to be modulated by oral hygiene practices rather than solely by systemic status.

## Introduction

Periodontal disease has been widely studied in relation to systemic illnesses including cardiac anomalies, diabetes mellitus (DM), obesity, and prediabetes^1–5^. This relationship is usually bidirectional with inflammation serving as the key underlying mechanism^5^. Prediabetes is clinically defined by hemoglobin A1c (HbA1c) values between 5.7% and 6.4%^6^. Studies^3,7^ have shown that prediabetic patients are more prone to periodontal inflammatory insults in contrast to normoglycemic individuals (HbA1c levels < 5.7%). Sustained HbA1c elevation, often observed among prediabetic patients, facilitates the deposition of advanced glycation end products (AGEs) in multiple body tissues, including periodontal tissues^8^. These endproducts elevate the production of destructive inflammatory cytokines such as interleukin-6 (IL-6) in periodontal fibroblasts^9,10^. This process, in turn, worsens periodontal inflammation in prediabetic individuals.^11^ On a similar note, obesity (body mass index [BMI] ≥ 30 Kg/m^2^) is a chronic metabolic disorder characterized by excessive adipose tissue accumulation, leading to systemic low-grade inflammation^12^. Adipose tissue acts as a metabolically active endocrine organ, characterized by the release of pro-inflammatory mediators including IL-6 and tumor necrosis factor-alpha (TNF-α), accompanied by a diminished secretion of anti-inflammatory adipokines^13^. This dysregulated cytokine profile enhances inflammation and impairs immune function, predisposing individuals to several comorbidities, including cardiac diseases, DM, prediabetes, and periodontitis^13–15^. However, despite the availability of such evidence^11,13^, existing studies have predominantly evaluated prediabetes and obesity as independent variables, with limited consideration of their combined or interactive effects on periodontal inflammation and whole salivary cytokine expression. The coexistence of obesity and prediabetes represents a clinically relevant phenotype characterized by amplified systemic inflammation, yet its specific impact on periodontal status and whole salivary IL-6 levels remains insufficiently elucidated in scientific literature. Furthermore, it is unclear whether the concurrent presence of these conditions exerts an additive or synergistic effect on periodontal tissue destruction and inflammatory biomarker profiles.

Unstimulated whole saliva (UWS) is a multifaceted biological fluid, which is essential for maintaining homeostasis through its functions in lubrication, digestion, antimicrobial protection, and immune modulation. Elevated levels of inflammatory mediators, including IL-6 and TNF-α, are commonly found in the UWS of individuals affected by periodontal disease compared with those without the condition^16–18^. In this context, UWS is a potential medium for assessment of biomarkers of periodontal and systemic inflammation. To date, no investigations have explored the association between periodontal health and salivary IL-6 concentrations in individuals with obesity, either with or without prediabetes. With this background, the objective was to investigate the association between periodontal status and whole salivary IL-6 levels in normal-weight and obese individuals with and without prediabetes. The *null* hypothesis is that no link exists between salivary IL-6 and periodontal status in normal-weight and obese individuals with and without prediabetes.

## Methods

### Ethical approval

The study protocol and research project were reviewed and approved by the Ethics Board of the College of Dentistry, King Saud University, Riyadh, Saudi Arabia (Approval No. E-25-9746). The investigation was conducted in accordance with the ethical principles outlined in the Declaration of Helsinki (2013 revision) and applicable institutional guidelines and regulations governing research involving human participants^19^. All participants were provided with a written explanation of the study objectives and procedures prior to enrollment. Written informed consent was obtained from all participants before inclusion. Participants were clearly informed that their participation was entirely voluntary, that they could withdraw at any time without penalty, and that refusal to participate would not affect their care. All individuals were encouraged to ask questions and seek clarification as needed before and during their participation.

### Study duration

The study was performed between November 2024 and October 2025.

### Patient selection

Inclusion criteria: (a) adults aged 18 years or older; (b) participants with clinically confirmed obesity (BMI ≥ 30 kg/m²)^20^; (c) individuals with medically diagnosed prediabetes (HbA1c 5.7%-6.4%)^6^; (d) self-reported systemically healthy normal weight individuals (BMI 18.5–24.9 kg/m²)^21^. Individuals with other self-reported diseases such as cardiac disorders, hepatitis, renal disorders, HIV/AIDS, and respiratory disorders were excluded. Those who had used corticosteroids, antibiotics, bisphosphonates, non-steroidal anti-inflammatory agents, or probiotics over the previous 90 days were excluded. Moreover, self-reported tobacco smokers or chewers, alcohol users, completely edentulous subjects, and/or patients who had undergone surgical or non-surgical periodontal therapy in the last three months were not considered eligible.

### Questionnaire

The questionnaire was administered to all participants by the principal investigator (MYS) and recorded the patient’s age, gender, and education status (ES) (school-level^22^, college-level^22^, or university-level^22^. The questionnaire also gathered information regarding the duration of prediabetes and obesity. Moreover, information regarding the family history of prediabetes and obesity and oral hygiene practices was obtained. Participants were also asked about the frequency at which they had dental checkups.

### Assessment of body mass index

A calibrated examiner (MYS; κ = 0.86) measured the participants’ BMI. Body weight was recorded using a digital weighing scale. Participants stood on the scale barefoot and wearing light clothing, ensuring accuracy to the nearest 0.1 kg. Standing barefoot in an upright posture, participants’ stature was recorded using a stadiometer (Seca^®^/Seca GmbH & Co.KG, Hamburg-Germany). Based on the BMI recorded, participants were categorized into normal weight (18.5–24.9 kg/m²), overweight individuals (BMI 25–29.9 kg/m²) and individuals with obesity (BMI ≥ 30 Kg/m^2^) individuals^21^.

### Assessment of healthcare records for hemoglobin A1c levels

The digital healthcare records of all participants were reviewed to obtain the most recent HbA1c levels. The timeframe since the last HbA1c measurement was also assessed. Participants with HbA1c < 5.7% and between 5.7 and 6.4% were considered normoglycemic and prediabetic, respectively^6^.

### Periodontal evaluation

Plaque index (PI)^23^ and gingival index (GI)^24^ were evaluated on the mesial, distal, buccal/facial, and palatal/lingual aspects. Probing depth (PD)^25^ and clinical attachment loss (CAL)^25^ were measured on mesiobuccal/facial, midbuccal/facial, distobuccal/facial, mesiolingual/palatal, midlingual/palatal and distolingual/palatal per tooth using a graded probe (UNC-15, Hu-Friedy Manufacturing Company, Chicago, IL USA). Marginal bone loss (MBL) was measured on digital radiographs taken using the long-cone paralleling technique^26^. PD and CAL were measured as the linear distance between the gingival margin and the bottom of the sulcus and from the cementoenamel junction (CEJ) to the base of the sulcus, respectively. The numbers of missing teeth (MT) were also recorded. All clinical and radiological investigations were performed by a calibrated investigator (MYS; κ = 0.88).

### Saliva sample collection and measurement of interleukin-6

The samples were collected as described in previous studies^11,14^. Saliva samples were collected between 7:00–9:00 am under standardized conditions to minimize diurnal variation. Participants avoided food, drink, and oral hygiene for at least one hour prior. Saliva was obtained by passive drooling for five minutes, with unstimulated whole salivary flow rate (UWSFR) recorded, and samples were kept on ice. Upon collection, samples were centrifuged at 3000 rpm for 10 min at 4 °C, and the supernatant was aliquoted and stored at − 80 °C. Salivary IL-6 levels were quantified using a high-sensitivity ELISA kit (Invitrogen, USA) according to manufacturer instructions. Samples and standards were tested in duplicate, with absorbance read at 450 nm and concentrations determined via a standard curve (detection range: 16–1690 pg/ml; sensitivity: <1 pg/ml). All procedures were conducted by a trained, calibrated, and blinded investigator (κ = 0.86). All samples were assessed for IL-6 levels within 90 days of collection.

### Sample-size estimation

An a priori sample-size calculation was performed using G*Power software (version 3.1.9.7; Heinrich-Heine-Universität Düsseldorf, Düsseldorf, Germany). Since the primary aim was to compare periodontal status across four independent study groups, probing depth (PD) was prespecified as the primary outcome and the calculation was based on a fixed-effects one-way ANOVA. A moderate-to-large effect size (Cohen’s *f* = 0.35) was assumed on the basis of pilot observations and previously published literature^11,13^ involving periodontal inflammatory parameters in individuals with metabolic abnormalities. Using a two-tailed significance level of 5% and a statistical power of 80%, the minimum required sample size was estimated at 84 participants (21 per group).

### Statistical analysis

All statistical analyses were performed using a commercial software package (IBM SPSS Statistics for Windows, version 28.0; IBM Corp., Armonk, NY, USA). The Shapiro-Wilk test was applied to evaluate the normality of data distribution. The Shapiro–Wilk test confirmed normal distribution for all assessed variables, including PD (W = 0.971, *p* = 0.128), CAL (W = 0.96, *p* = 0.09), MBL (W = 0.95, *p* = 0.071), and salivary IL-6 levels (W = 0.98, *p* = 0.312). Descriptive analyses were expressed as mean ± standard deviation for continuous variables and as frequencies with corresponding percentages for categorical variables. Group comparisons were carried out using one-way ANOVA, followed by Bonferroni post-hoc testing where appropriate. Correlation analyses were undertaken using Pearson’s or Spearman’s coefficients to explore associations between salivary IL-6 concentrations and periodontal clinical as well as radiographic indices. To further examine the independent contribution of periodontal health to salivary IL-6 levels, linear regression/correlation models were constructed. Statistical significance was defined at a threshold of *p* < 0.05.

## Results

### Demographics of the study population

**Fig. 1:**
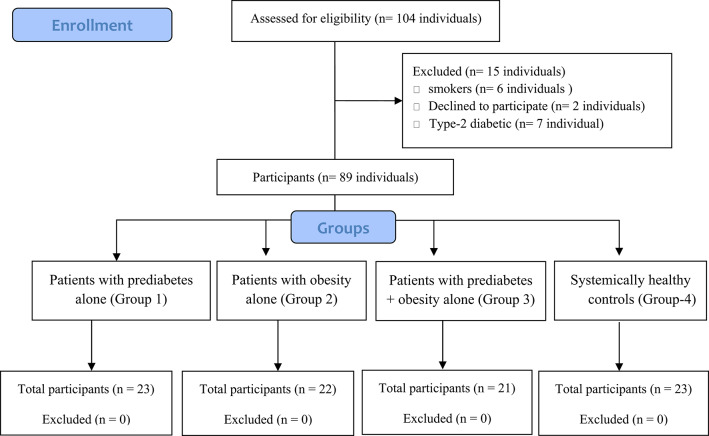
STROBE flow diagram.

One hundred and four individuals were assessed for eligibility. Fifteen individuals were excluded. In total, 89 individuals were included (Fig. 1). Participants were divided into the following groups based on their BMI and HbA1c levels: individuals with prediabetes alone (*n* = 23), individuals with obesity alone (*n* = 22), prediabetic individuals with obesity (*n* = 21), and systemically healthy individuals (*n* = 23). Patients with prediabetes (Groups 1 and 3) exhibited significantly higher hemoglobin A1c levels compared with systemically healthy individuals (Group 4). Similarly, participants with obesity (Groups 2 and 3) demonstrated significantly greater BMI than both prediabetic individuals without obesity (Group 1) and healthy controls (Group 4).Regarding oral health behaviors, toothbrushing frequency was lowest in individuals with combined prediabetes and obesity (Group 3), who reported significantly poorer oral hygiene practices compared to all other groups, particularly those who were systemically healthy (Group 4), who consistently demonstrated the most favorable behaviors. Dental attendance patterns also differed: the interval since the most recent dental visit was significantly longer in the prediabetes with obesity group (Group 3) compared to the other groups (Table 1).

**Table 1:** Demographic characteristics, systemic health status, and oral health-related behaviors among study groups stratified by glycemic status and obesity.

Parameters	Patients with prediabetes alone (Group 1)	Patients with obesity alone (Group 2)	Patients with prediabetes and obesity (Group 3)	Systemically healthy individuals (Group 4)
Participants (n)	23	22	21	23
Gender	12 males: 11 females	16 males: 6 females	12 males: 9 females	11 males; 12 females
Age in years	51.7 ± 5.5 years	53.2 ± 4.6 years	52.9 ± 5.2 years	53.1 ± 5.6 years
Hemoglobin A1c (%)	6.1 ± 0.2%^*^	5.4 ± 0.2%	6.2 ± 0.2%^*^	4.6 ± 0.2%
Body mass index (Kg/m^2^)	21.1 ± 1.8 Kg/m^2^	36.1 ± 3.1 Kg/m^2†^	37.3 ± 2.5 Kg/m^2†^	22.2 ± 1.5 Kg/m^2^
Duration of prediabetes	2.8 ± 0.4 years	NA	5.3 ± 0.7 years	NA
Duration of obesity	NA	4 0.1 ± 1.6 years	5.5 ± 0.5 years	NA
Education status				
School-level	NA	NA	6 (28.6%)	NA
College level	3 (13%)	7 (31/8%)	12 (57.1%)	NA
University-level	20 (87%)	15 (68.2%)	3 (14.3%)	23 (100%)
Family history of hyperglycemia	10 (43.5%)	13 (59.1%)	15 (71.4%)	NA
Family history of obesity	4 (17.4%)	10 (45.5%)	11 (52.4%)	NA
Toothbrushing (twice daily)	19 (82.6%)	16 (72.7%)	4 (19%)	23 (100%)
Interproximal flossing (at least once daily)	8 (34.8%)	4 (18.2%)	NA	12 (52.2%)
Most recent dental visit	0.81 ± 0.3 years ago	1.1 ± 0.4 years ago	3.2 ± 0.4 years ago	0.86 ± 0.2 years ago

Data are presented as mean ± standard deviation for continuous variables and as percentage for categorical variables. NA: Not applicable ^*^Compared with Group 4 (*P* < 0.05) ^†^Compared with groups 1 (*P* < 0.05) and 4 (*P* < 0.05).

### Periodontal parameters, saliva collection and IL-6

The PI (*P* < 0.01), GI (*P* < 0.01), CAL (*P* < 0.01), PD (*P* < 0.01), mesial (*P* < 0.01) and distal (*P* < 0.01) MBL, and numbers of MT (*P* < 0.01) were significantly higher among individuals in Group 3 compared with individuals in groups 1, 2 and 4 (Table 2). The UWSFR and IL-6 concentrations were higher in Group 3 (*P* < 0.01) than groups 1, 2, and 4 (Table 3).

**Table 2:** Comparison of clinical and radiographic periodontal parameters across study groups.

Parameters	Patients with prediabetes alone (Group 1)	Patients with obesity alone (Group 2)	Patients with prediabetes and obesity (Group 3)	Systemically healthy individuals (Group 4)
Plaque index	0.4 ± 0.06^*^	0.44 ± 0.08^*^	0.88 ± 0.1^†^	0.33 ± 0.05
Gingival index	0.45 ± 0.08^*^	0.5 ± 0.06^*^	0.91 ± 0.08^†^	0.39 ± 0.08
Clinical attachment loss (mm)	1.8 ± 0.2 mm^*^	1.9 ± 0.2 mm^*^	4.5 ± 0.1 mm^†^	1.2 ± 0.07 mm
Probing depth (mm)	2.9 ± 0.8 mm^*^	3.2 ± 0.3 mm^*^	5.7 ± 0.2 mm^†^	2.5 ± 0.09 mm
Marginal bone loss (mesial)	2.6 ± 0.08 mm^*^	3.1 ± 0.9 mm^*^	4.6 ± 0.08 mm^†^	2.1 ± 0.2 mm
Marginal bone loss (distal)	2.5 ± 0.1 mm^*^	3.06 ± 0.7 mm^*^	4.5 ± 0.1 mm^†^	2 ± 0.09 mm
Missing teeth (n)	2 ± 0.2 teeth^*^	1.8 ± 0.08 teeth^*^	6.7 ± 0.5 teeth^†^	1.1 ± 0.06 teeth

Data are expressed as mean ± SD. ^*^Compared with Group 3 (*P* < 0.01) ^†^Compared with Group 4 (*P* < 0.01) mm: millimeters.

**Table 3:** Unstimulated whole salivary flow rate and interleukin-6 concentrations among study groups.

Parameters	Patients with prediabetes alone (Group 1)	Patients with obesity alone (Group 2)	Patients with prediabetes and obesity (Group 3)	Systemically healthy individuals (Group 4)
Unstimulated whole salivary flow rate (ml/min)	0.32 ± 0.04 ml/min^*^	0.33 ± 0.07 ml/min^*^	0.14 ± 0.05 ml/min^†^	0.35 ± 0.05 ml/min
Interleukin-6 levels (pg/ml)	24.3 ± 4.9 pg/ml^*^	26.3 ± 6.1 pg/ml^*^	252.1 ± 81 pg/ml^†^	22.5 ± 3.9 pg/ml

Data are expressed as mean ± SD. ^*^Compared with Group 3 (*P* < 0.01) ^†^Compared with Group 4 (*P* < 0.01) pg/ml: picograms per milliliter ml/min: milliliters per minute.

### Linear regression analysis

**Fig. 2:**
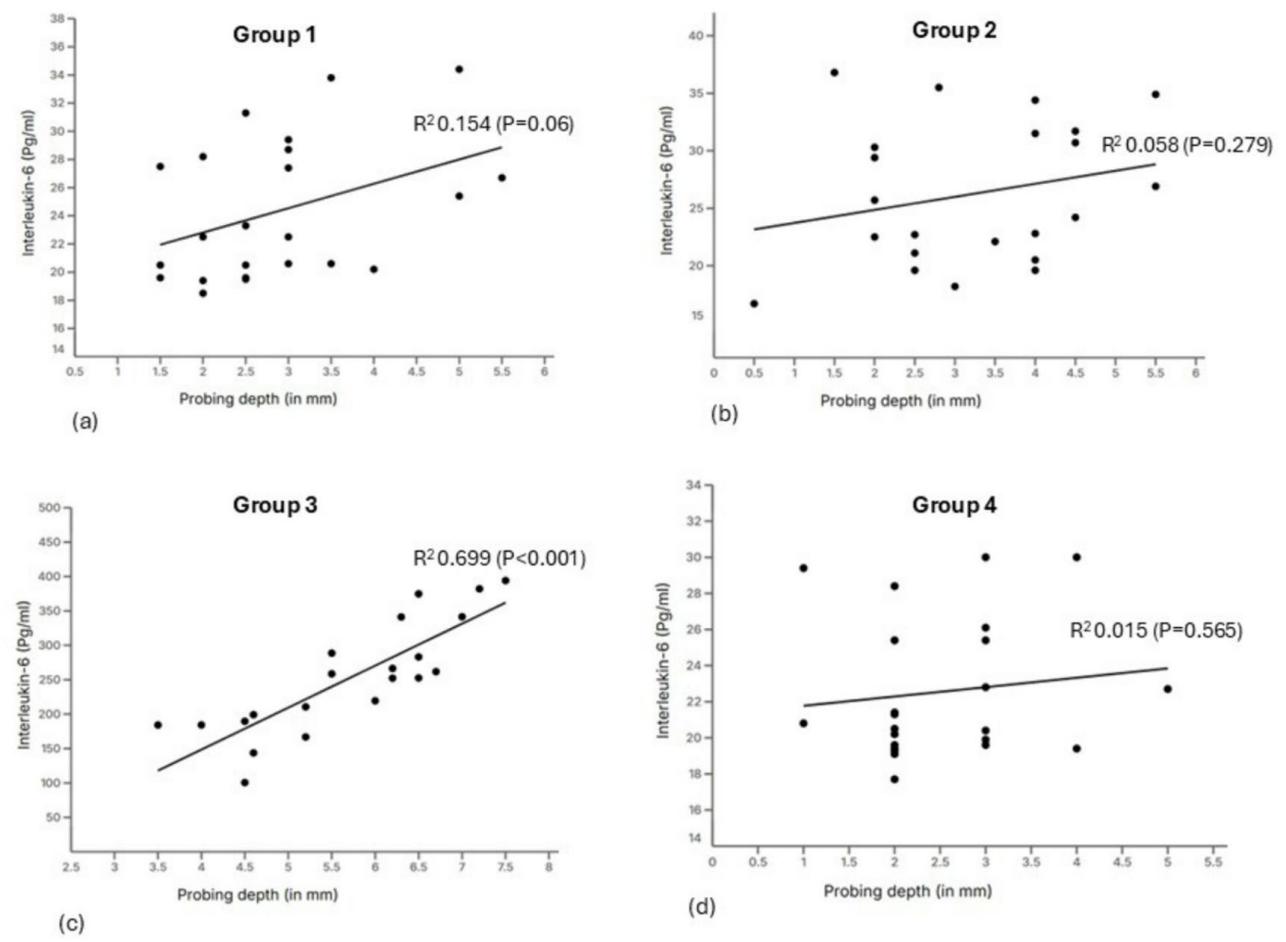
Scatter plot demonstrating the correlation between probing depth (PD) and whole salivary interleukin-6 (IL-6) concentrations.

A statistically significant correlation was found between PD and whole salivary IL-6 levels in Group 3 (Fig. 2). In all groups, there was no correlation between duration of prediabetes and obesity, age, UWSFR, GI, gender, BMI, PI, CAL, MBL, MT, family history of hyperglycemia and/or BMI, OHM protocols, and most recent visit to a dentist (data not shown).

## Discussion

Both obesity and prolonged hyperglycemia, commonly seen in prediabetes and uncontrolled DM, are well-documented contributors to the onset and advancement of periodontal disease such as periodontitis^3,14,27–30^. An explanation that is often posed in this regard is that hyperglycemia enhances the production of AGEs that worsen periodontal inflammation by inducing a state of oxidative stress and promoting the production of destructive inflammatory cytokines in periodontal tissues^31,32^. Likewise, obesity alters the diversity of oral microbiota and induces dysregulation in the immune system thereby indicating a possible relationship between the latter and periodontal diseases^33^. Hence, it was hypothesized that prediabetic patients with obesity would exhibit greater periodontal inflammation and higher salivary IL-6 concentrations compared with healthy controls. Interestingly, our results showed no significant difference in periodontal parameters in groups 1 and 2. Several factors appear to have influenced the results of the present investigation. Notably, the mean age of participants in Groups 1 and 2 was approximately 50 years. Increasing age is associated with a heightened susceptibility to periodontal disease, with individuals aged 60 years or older exhibiting more pronounced periodontal inflammation compared to younger counterparts^34^. Scientific evidence shows that individuals with a longer history of type-2 DM (≥ 5 years) demonstrate more severe periodontal inflammation in contrast to individuals with a shorter duration of type-2 DM^35^. However, in the present investigation, the duration of prediabetes among patients in groups 1 and 3 was relatively short. Therefore, it is challenging to establish a true relationship between periodontal inflammation and prediabetes. Further longitudinal studies are needed to assess the effect of the duration of prediabetes and obesity on the severity of periodontal inflammatory parameters. Moreover, participants in Group 3 had significantly high whole salivary IL-6 levels and demonstrated significantly higher scores of clinical and radiographic periodontal inflammatory parameters in contrast to other groups. It is therefore hypothesized that the cumulative inflammatory influence of prediabetes and obesity augmented periodontal inflammation and whole salivary IL-6 levels among participants in Group 3 compared with individuals with prediabetes and obesity alone.

A vigilant assessment of the results showed that routine OHM was superior among patients in groups 1, 2 and 4 in contrast to individuals in Group 3. Moreover, the ES of individuals in the former groups was superior, with most of the participants having attained university-level education in contrast to the latter group (Group 3). It has been reported that individuals with an underprivileged socioeconomic and ES are more susceptible to periodontal inflammation than individuals with a superior income and ES^34^. The authors speculate that a privileged ES positively influence OHM through multiple behavioral and cognitive, and socio-economic mechanisms such as a better comprehension of the importance of OHM, the consequences of inadequate care, and routinely visiting oral healthcare providers for check-ups and dental prophylaxis. Moreover, literacy may also translate into proactive behaviors, such as daily toothbrushing with interproximal flossing. It is pertinent to mention that individuals in Group 3 had last visited an oral healthcare provider over three years ago, were not flossing their teeth, and nearly 80% were brushing their teeth once daily. These factors could also have contributed to the elevated whole salivary IL-6 levels and compromised clinical and radiographic periodontal status among participants in Group 3 compared with individuals in groups 1, 2 and 4. It is recommended that patients with systemic disease, including hyperglycemia and BMI-related disorders, should be educated about the relationship between oral and systemic health and the significance of oral and systemic health maintenance towards an overall superior quality of life.

The findings of this study support the hypothesis that obesity in conjunction with a prediabetic state significantly contributes to a pro-inflammatory oral environment than prediabetes and obesity alone, as evidenced by increased IL-6 levels in UWS. By no means is this inflammatory cytokine a diagnostic parameter for prediabetes, obesity and/or periodontal disease; however, whole salivary IL-6 seems to be a potential biomarker of the ongoing inflammatory response in these individuals. Interestingly, whole salivary IL-6 levels were significantly correlated with PD among patients in Group 3, with no significant correlations observed in the other groups. Moreover, elevated salivary IL-6 may exacerbate periodontal inflammation through immune dysregulation and enhanced tissue destruction.

### Limitations

The cross-sectional design of this study inherently restricts the ability to establish causal relationships between periodontal parameters and salivary IL-6 levels. Therefore, the observed associations should be interpreted with caution, as they do not imply temporality or directionality. Moreover, participants were not classified according to standardized periodontal diagnostic criteria (such as staging and grading of periodontitis). Instead, periodontal status was evaluated using continuous clinical and radiographic parameters such as PD, CAL and MBL. While this approach allowed for quantitative assessment of periodontal inflammation, the absence of categorical periodontal diagnoses limits the ability to generalize the findings within established diagnostic frameworks and compare outcomes directly with studies using contemporary classification systems. Longitudinal and interventional studies with larger and more heterogeneous cohorts are warranted to confirm these findings and to better elucidate potential causal pathways linking periodontal diagnosis, obesity, and prediabetic status. Another limitation is that gingival recession and gingival margin position were not explicitly recorded. Although PD and CAL were assessed using standard clinical definitions, the absence of direct measurements of gingival margin levels limits the interpretation of the relationship between PD and CAL. It remains unclear whether variations between these parameters were influenced by gingival enlargement or subtle recession. Dyslipidemia is a well-recognized component of metabolic disorders, including obesity and prediabetes^36,37^; and has been implicated in the pathogenesis of periodontitis^36,37^. It is therefore suggested that future studies should incorporate a comprehensive assessment of gingival margin position to allow a more precise characterization of periodontal tissue changes; and simultaneously assess systemic lipid profiles (such as total cholesterol, low- and high-density lipoproteins, and triglycerides) to attain a clearer understanding of the relationship between periodontal disease and obesity. Furthermore, it is noteworthy that although the sample size was determined a priori using a power calculation and met the minimum required threshold, it remains relatively modest, which may limit the external validity and generalizability of the results to broader populations with diverse demographic and clinical characteristics.

## Conclusions

Prediabetes in the presence of obesity is associated with an increased burden of periodontal inflammation; however, this relationship appears to be modulated by oral hygiene practices rather than solely by systemic status. Effective OHM may mitigate periodontal deterioration even in metabolically compromised individuals. These findings highlight oral hygiene as a key modifiable factor in reducing periodontal inflammation in this population.

## Data Availability

Data is available on reasonable request (Contact: Dr. Marwa Y. Shaheen).
